# Patterns and trends of medical student research

**DOI:** 10.1186/1472-6920-13-175

**Published:** 2013-12-28

**Authors:** Dakshitha Praneeth Wickramasinghe, Chamila Sudarshi Perera, Supun Senarathna, Dharmabandhu Nandadeva Samarasekera

**Affiliations:** 1 Department of Clinical Sciences, Faculty of Medicine, Sir John Kotelawala Defence University, Colombo, Sri Lanka; 2 Department of Surgery, Faculty of Medicine, University of Colombo, Kynsey road, Colombo 08, Sri Lanka

**Keywords:** Medical student, Research, Patterns, Trends

## Abstract

**Background:**

Our study describes the change in the research output, trends and content of published research involving medical students over the last century.

**Methods:**

Pubmed® and Scopus® were searched for keywords ‘Medical Student’ in the affiliation field. The search results were combined in Endnote® and duplicate entries removed and the multiple variables described below were assessed.

**Results:**

The combined searches after excluding duplicates yielded 416 results and 66 articles were excluded. There was an exponential increase in medical student research from 1980–2010. Medical student was the first author in 170 (48.6%) studies and 55 studies were authored by a single medical student. The 3 most common areas of research in descending order were Psychiatry (n = 26, 7.4%), General Medicine (n = 24, 6.9%) and Medical Education (n = 21, 6%). The commonest type of articles, in descending order were review articles (n = 48, 13.7%), Cross sectional studies (n = 47, 13.4%) and Case reports (n = 43, 12.3. The majority of these articles (n = 207, 59.1%) have never been cited subsequently. The trend of increasing number of articles was seen equally among all article types, fields and countries.

**Conclusions:**

There is an exponential increase in articles by medical students but the majority of articles have not been cited. The numbers of medical student authors per publication have remained static while the total numbers of authors have increased. The proportions in the type of articles, fields of study and country of origin have largely remained static. Publishers and authors should strive to enhance the quality and quantity of data available in indexing services.

## Background

There is an increasing trend to encourage scientific research all over the world, both in the East as well as the West. Medical students also constitute a significant proportion among medical professionals engaged in research, though this contribution is less compared to students of other professions [1]. Research provides the students with an intellectually challenging, self-learning experience [2], and participating in research is important in producing doctors with an understanding of evidence-based medicine. Participation in research and audits while in medical school can help develop these skills whilst prompting interest in academic pursuits [1].

There are many examples in the history where medical students’ contribution has lead to new interventions in medicine. The discovery and purification of insulin was made by the researcher Frederick Banting and his second-year medical student assistant Charles Best [3], whilst the discovery of the anticoagulant Heparin was made by Jay Mclean, a medical student working at the John Hopkins University [1].

Medical students’ involvement in research is a longstanding tradition and has been an integral component of medical education for years. Research experience helps foster scientific thought and nurture evidence-based practice in clinical settings [4]. Many medical institutions in the world are encouraging students to engage in scientific research from first year itself, by various methods and the trend is on the rise [4]. In some countries in the world, research experience as a medical student has become a compulsory component of the medical degree. However, most motivation for engaging in research appears to be largely curriculum vitae driven [1]. Furthermore, experience of research at medical school has been shown to promote medical student interest in academic medical careers and postgraduate research productivity [5]. Without experience in research, in Germany graduating medical students are unable to assume the title of doctor. In the United Kingdom, the General Medical Council guidelines ‘Tomorrow’s Doctors’ recognize the importance of critical evaluation of information as an essential skill that all doctors should possess [6]. A number of studies have shown that students who become involved with research while still in medical school have superior postgraduate research productivity [7].

Though the importance of medical student research has been long been identified, there is scant evidence on patterns and trends in medical student research. The primary aim of this study was to find the patterns and trends of medical student research. Secondary aims were to review the impact/relevance of these publications and to identify any academic or ethical concerns regarding medical student research.

## Method

Pubmed® and Scopus® were searched for the key words ‘medical student’ in the author affiliation field. No other keywords or restrictions were used. Pubmed® search was done using Endnote® and the results from the Scopus® search was imported into the same Endnote® database. The combined database was then scanned for duplicates, first using the inbuilt function in Endnote® and then manually to ensure the quality of the database. Two investigators (CP and SS) then scrutinized the abstracts available. They first checked that at least one medical student was involved and the incorrect entries were removed. Then they reviewed each abstract and/or the full text article to find the year of publication, number of authors, number of medical students as authors, the academic year of medical student authors, the country of origin of the article, specialty, article type and number of citations. Studies which neither had the abstract nor the full text articles available were removed from the database. Studies in which there were no details about medical student involvement were scrutinized with available data and were excluded if the involvement of medical students was not explicitly disclosed. Studies that only had non-medical students involved were also excluded.

Google scholar® was not used to check for citations because it includes references by non-indexed journals and website as citations. It was also not included in identification of articles because it did not allow search using author affiliation.

The data was then entered into a SPSS database (IBM SPSS Statistics, SPSS Inc., Chicago IL). Continuous data were analyzed using the mean and standard deviation and histograms were created to identify trends.

## Results

The searches when combined in Endnote® yielded 416 unique abstracts. 60 articles were excluded since they did not include a medical student as an author and a further 6 were excluded because of lack of available data. The first entry was in 1933. There were 44 abstracts in the first 4 months of 2012, only second to the 77 abstracts in 2011 and equal to the abstracts in 2010.

**Figure 1:**
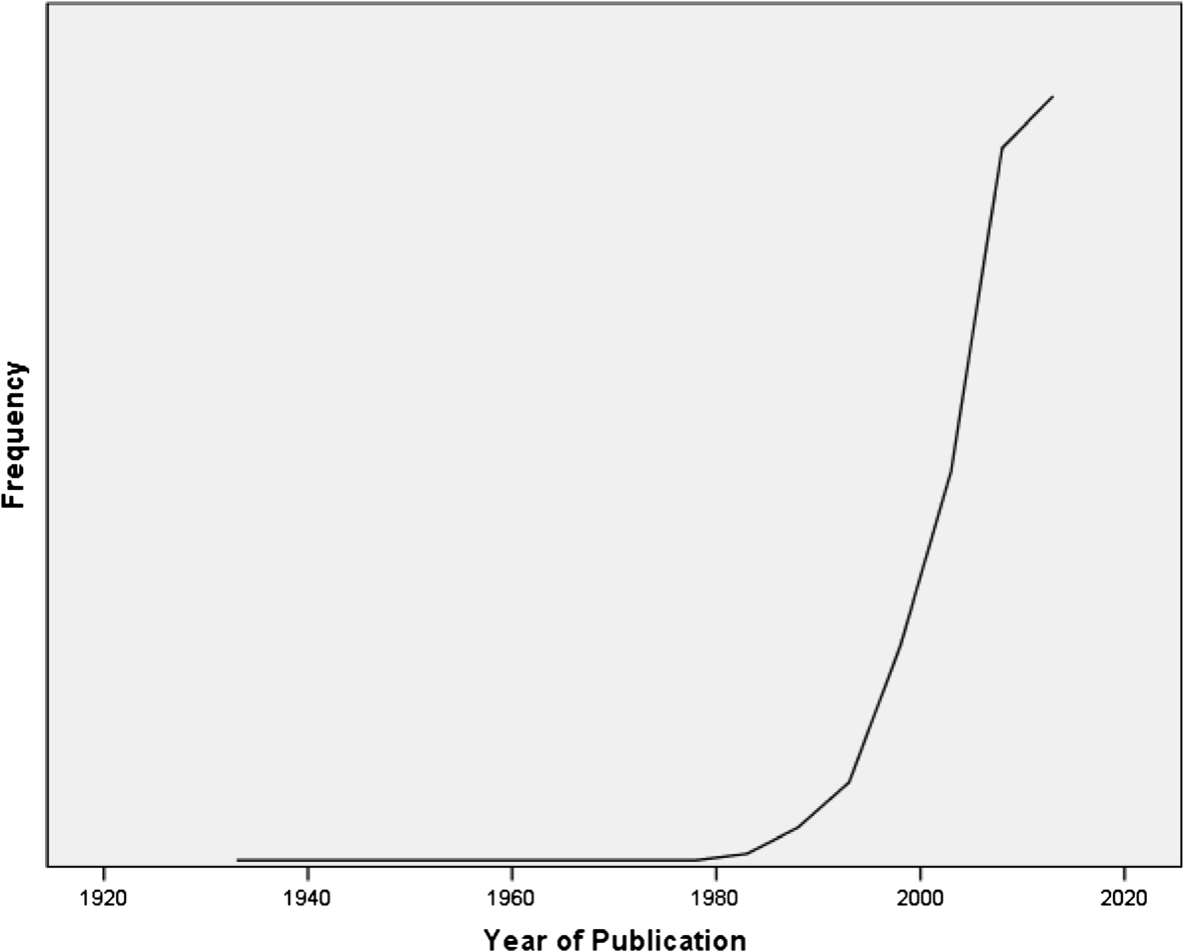
The number of publications by year.

There was an exponential increase in medical student research over the latter part of the last century and the first part of this century, the increase becoming marked from around 1980 onwards (Figure 1). In the majority of articles (74%), there was a single medical student as either the first author (n = 170, 48.6%) or the 2nd author (n = 89, 25.4%).55 (15.7%) studies were authored by a single medical student. On average, 44% of authors were medical students. However, the majority (n = 256) of articles only had 1 medical student. There was an increase in the number of authors per article throughout the study period (mean ± SD; pre 1990 = 1.92 ± 1.3, 1990-1999 = 3.8 ± 2.4, 2000–2009 – 4.05 ± 2.6, 2010 onwards = 5.05 ± 2.7, Kurskall Wallis test H = 31.6, 1 d.f., P < 0.0001). However, the number of medical student authors per article remained remarkably static (mean ± SD; pre 1990 = 1.2 ± 0.5, 1990-1999 = 1.4 ± 1.0, 2000–2009 – 1.4 ± 1.2, 2010 onwards = 1.7 ± 1.6, Kurskall Wallis test H = 3.4, 1 d.f., P = 0.32).

The 5 most common areas of research that included medical students, in descending order, were Psychiatry (n = 26, 7.4%), General Medicine (n = 24, 6.9%), Medical Education (n = 21, 6%), Oncology (n = 20, 5.7%) and Community Medicine (n = 18, 5.1%). The commonest type of articles, in descending order were review articles (n = 48, 13.7%), cross sectional studies (n = 47, 13.4%), case reports (n = 43, 12.3%), case control studies (n = 41, 11.7%) and cohort studies (n = 37, 10.6%). A majority of these articles (n = 207, 59.1%) had not been cited at least once and the mean number of citations were 4.5 ± 12.5.

**Figure 2:**
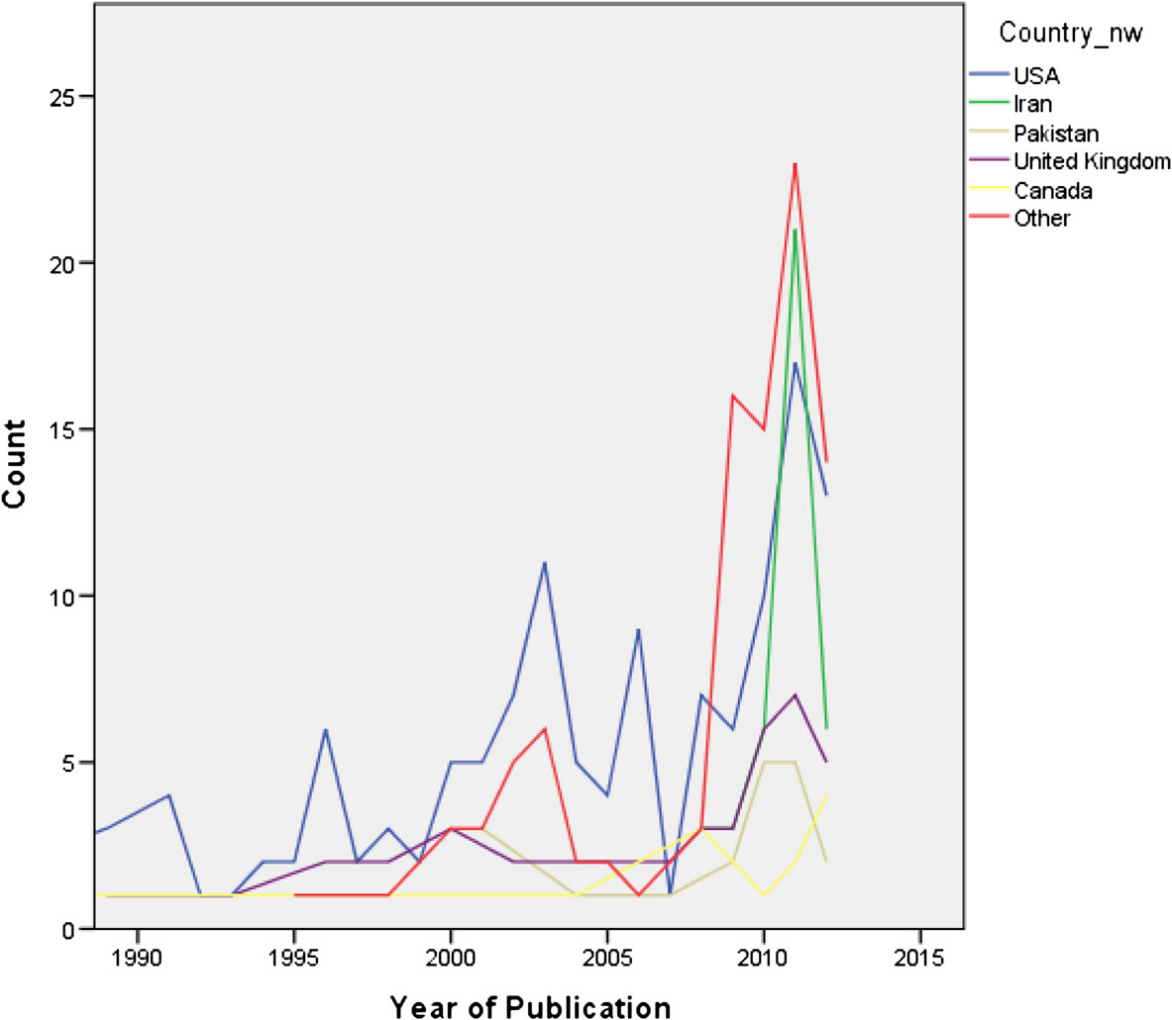
The number of publications by year, by country.

**Figure 3:**
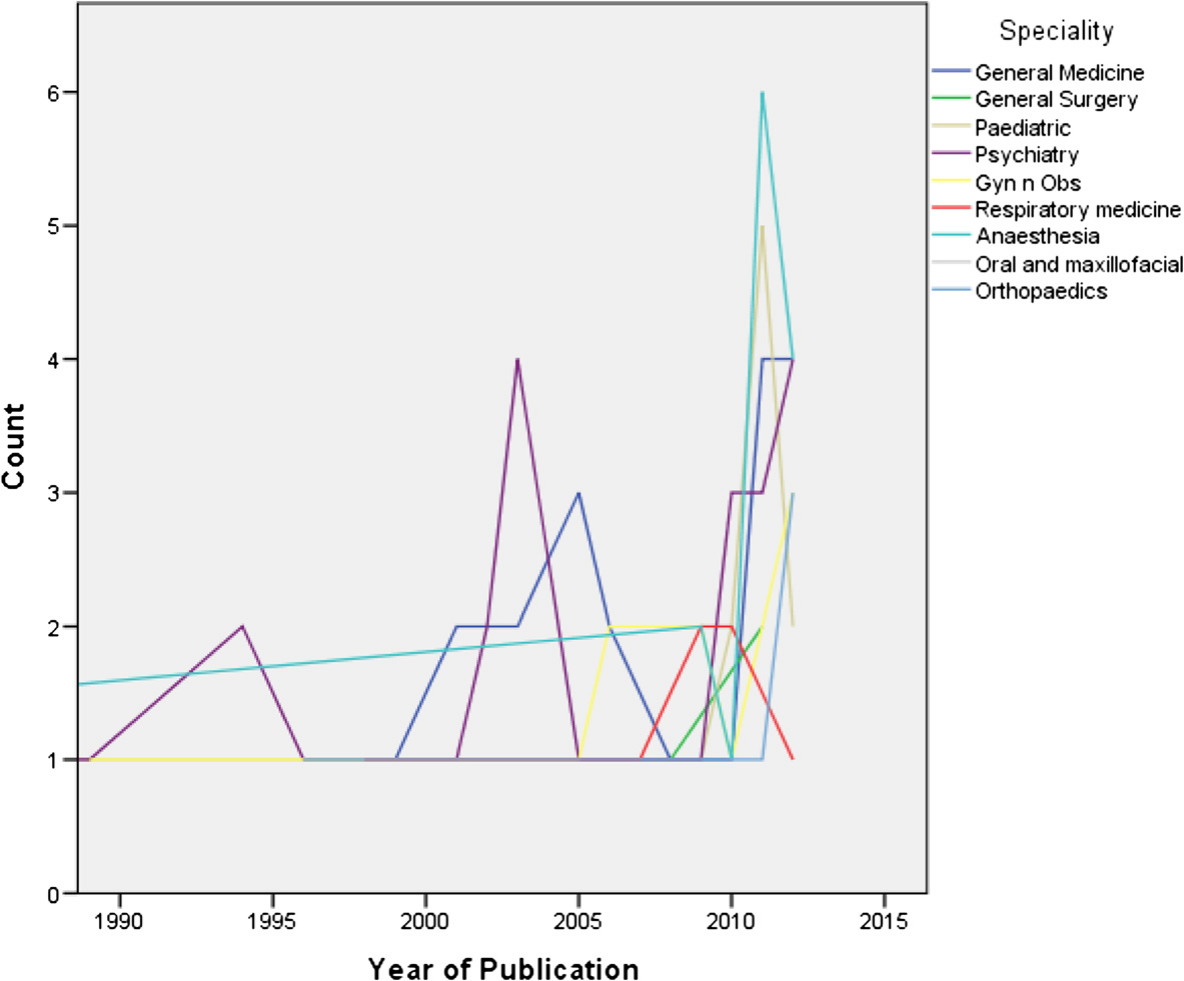
The number of publications by year, by type of content area.

The trend of increasing number of articles each year was seen equally among all article types, fields and countries (Figures 2 and 3).

## Discussion

Our findings have identified an exponential increase in medical student research, especially evident since 1980 and this increase is similar to the overall increase in medical research publications during the same period [8]. There are numerous articles originating from the west [9-12] as well as the East [13-16] describing the increased interest in medical student research. A recent study done in Brazil [17] identified that only 7% of students expressed no interest in research and Sanford et al. [18] reports that 24% of students in his institution were co-authors in articles. There are several possible reasons for this increase; i.e. increased attempts by state and institutions to introduce medical students to research [5,19-21], earlier involvement in research [18], exposure to well established programs often geared for medical student schedules [19,20], financial assistance [22] and higher access to mentors [22]. What is perhaps unusual is that the ratio between different article types (e.g.- case reports, randomized trials, reviews) have not significantly changed. Druss et al. [8] reports a shift from basic science research to clinical research over the last 20 years of the 20th century.

Multiple authors have also reported the increase in the mean number of authors per article [8,23]. The multiple authorship could possibly arise from an increased tendency of multidisciplinary research, though some authors refute this claim [23]. However, we have identified that the mean number of medical student authors have not increased proportionately. This raises a serious concern and that is, are senior authors ‘using’ medical students to increase their tally of publications? Several authors have identified similar issues previously in publications. Among articles published, there are a large number of ‘honorary authors’ [24] and we may be seeing an increase of a similar trend. Shapiro et al. [25] claims that ‘The two core purposes of scientific authorship to confer credit and denote responsibility for research are not adequately being met by these authorship practices’ and has even proposed a revision to the Vancouver convention. The Vancouver convention was developed by the International Committee of Medical Journal Editors (ICMJE) and states that there need to be “substantial contributions” by an individual for either conception and design, data acquisition or analysis and interpretation for authorship to be awarded.

The implications of this increasing interest in research are great. The potential benefits to students are closer mentorship by individual faculty, enhanced capabilities in interpretation of research findings and increased confidence to assess conditions encountered in clinical care [26]. Society may also benefit by having physicians available to create and apply new knowledge related to biomedicine [26]. The relationship with the mentor often goes beyond advising on research [27] and thus there may be improvement in other academic and non academic aspects as well. Research trains individuals to gather information, assess them objectively and to make clear decisions, all of which are important in clinical decision-making and patient care. Doctors who have received scientific training during their medical education are at an advantage when it comes to decision making [28]. There is also evidence to suggest that doctors who have participated in scientific programs makes better professional decisions and diagnosis [29]. The positive impact on the motivation of medical students has also been well established [5,30-32].

Our findings identified 133 articles from the USA, 40 from the UK and only 86 from 10 Asian countries combined. We do not have sufficient data to elucidate a reason, and further research is necessary to identify whether it is a lack of financial or institutional support, lack of motivation or a lack of the necessary skills. However, Burgoyne et al. [33] reported a weakness in all domains of research skills among Asian medical students in Ireland compared to students from the USA or the UK. Smith et al. [34] in a study carried out in the USA also reports a higher publication rate among Caucasians when compared to Asians. Perhaps one reason for this is that Medicine tends to be a first degree in almost all students in Asian countries but tends to be a second degree for a considerable proportion of students in the USA or UK. Siemens et al. [35] reported that students with a previous degree have a better understanding of research methodology.

It is prudent that developing countries adopt the policies described above to encourage more research output from medical students. Encouraging medicine as a second degree to allow maturity is also an interesting alternative.

What is worrying is that these articles have been cited only rarely and the majority has never been cited. We are unable to objectively assess the scientific validity of each of these articles and their merits of citations. The Chronicle of Higher Education reports [36] that, only 45 percent of the articles published were cited within the first five years after publication and states, ‘while brilliant and progressive research continues apace here and there, the amount of redundant, inconsequential, and outright poor research has swelled in recent decades, filling countless pages in journals and monographs’ [36].

Our sample is by no means complete, which we consider as the biggest limitation in this study. A study done in the Netherlands identified around 50 articles by medical students indexed on Web of Science® over a 3 year period preceding 2007 [21]. Our search did not include this index, but our searches yielded only 12 articles from Netherlands from 2002 – 2012. However, we are confident that our sample is representative of the articles published and therefore provides valuable insight into the patterns and trends of medical student research though it may not be as robust when absolute numbers are concerned. A search using medical student journals would also be an alternative, but if all journals (indexed and non-indexed) were to be included, the process would probably prove exhaustive.

## Conclusions

There is an increasing number of medical student authored articles being published and the trend seems to be continuing. The number of medical student authors have remained static while the total number of authors have increased, raising valid concerns. The proportions in the type of articles, fields of study and country of origin have largely remained static throughout the study period. Publishers and authors should strive to enhance the quality and quantity of data available in indexing services. Therefore, we would like to encourage publishers and authors to ensure proper information on all fields, including the affiliation field when indexing articles, which would enable analysis that is more comprehensive.

## Competing interests

None of the authors have any financial or non-financial competing interests.

## Authors’ contributions

DPW and DNS were involved in the design of the study. CP and SS were involved in collecting and tabulating the data. All authors were involved in the analysis of the data and writing and reviewing the article. All authors read and approved the final manuscript.

## Pre-publication history

The pre-publication history for this paper can be accessed here:

http://www.biomedcentral.com/1472-6920/13/175/prepub
